# Reliability of Google Trends: Analysis of the Limits and Potential of Web Infoveillance During COVID-19 Pandemic and for Future Research

**DOI:** 10.3389/frma.2021.670226

**Published:** 2021-05-25

**Authors:** Alessandro Rovetta

**Affiliations:** ^1^ Research and Disclosure Division, Mensana srls, Brescia, Italy; ^2^ Technological and Scientific Research, Redeev srl, Napoli, Italy

**Keywords:** google trends, google trends data analysis, google trends data, COVID-19, social science research

## Abstract

**Background:** Alongside the COVID-19 pandemic, government authorities around the world have had to face a growing infodemic capable of causing serious damages to public health and economy. In this context, the use of infoveillance tools has become a primary necessity.

**Objective:** The aim of this study is to test the reliability of a widely used infoveillance tool which is Google Trends. In particular, the paper focuses on the analysis of relative search volumes (RSVs) quantifying their dependence on the day they are collected.

**Methods:** RSVs of the query *coronavirus* + *covid* during February 1—December 4, 2020 (period 1), and February 20—May 18, 2020 (period 2), were collected daily by Google Trends from December 8 to 27, 2020. The survey covered Italian regions and cities, and countries and cities worldwide. The search category was set to all categories. Each dataset was analyzed to observe any dependencies of RSVs from the day they were gathered. To do this, by calling i the country, region, or city under investigation and *j* the day its *RSV* was collected, a Gaussian distribution $X_{i}=X\left(\sigma_{i},{\bar{x}}_{i}\right)$ was used to represent the trend of daily variations of $x_{ij}=RSVs_{ij}$. When a missing value was revealed (anomaly), the affected country, region or city was excluded from the analysis. When the anomalies exceeded 20% of the sample size, the whole sample was excluded from the statistical analysis. Pearson and Spearman correlations between RSVs and the number of COVID-19 cases were calculated day by day thus to highlight any variations related to the day RSVs were collected. Welch’s t-test was used to assess the statistical significance of the differences between the average RSVs of the various countries, regions, or cities of a given dataset. Two RSVs were considered statistical confident when t<1.5. A dataset was deemed unreliable if the confident data exceeded 20% (confidence threshold). The percentage increase Δ was used to quantify the difference between two values.

**Results:** Google Trends has been subject to an acceptable quantity of anomalies only as regards the RSVs of Italian regions (0% in both periods 1 and 2) and countries worldwide (9.7% during period 1 and 10.9% during period 2). However, the correlations between RSVs and COVID-19 cases underwent significant variations even in these two datasets (Max |Δ| = + 625% for Italian regions, and Max |Δ|= +175%  for countries worldwide). Furthermore, only RSVs of countries worldwide did not exceed confidence threshold. Finally, the large amount of anomalies registered in Italian and international cities’ RSVs made these datasets unusable for any kind of statistical inference.

**Conclusion:** In the considered timespans, Google Trends has proved to be reliable only for surveys concerning RSVs of countries worldwide. Since RSVs values showed a high dependence on the day they were gathered, it is essential for future research that the authors collect queries’ data for several consecutive days and work with their RSVs averages instead of daily RSVs, trying to minimize the standard errors until an established confidence threshold is respected. Further research is needed to evaluate the effectiveness of this method.

## Introduction

A novel coronavirus was identified in Wuhan (Hubei province, China) in late 2019 (Wu et al., 2020). This was responsible for a severe respiratory disease named COVID-19 by the WHO on February 11, 2020. The virus, formerly reported as 2019-nCoV, was subsequently named SARS-CoV-2. Despite attempts by governments and the scientific community to contain the infection, COVID-19 has spread beyond the Chinese borders and was declared a pandemic by the WHO on March 11, 2020 (Cascella et al., 2021). Such pandemic has put a strain on health systems and economies of countries worldwide, causing more than 3 million deaths and forcing governments to implement very restrictive lockdowns (Askitas et al., 2021). In this scenario, fake news and inaccurate information circulated widely on the web creating severe issues to public health and economy all over the world (Pennycook et al., 2020; Rovetta and Bhagavathula, 2020; Tagliabue et al., 2020; Tasnim et al., 2020). Dr Tedros Adhanom Ghebreyesus-director of the World Health Organization (WHO) - claimed that the battle we are fighting does not only concern the epidemic but also its infodemic (UNS, 2020). Moreover, the WHO itself has launched an international campaign called “Managing the COVID-19 infodemic: Promoting healthy behaviors and mitigating the harm from misinformation and disinformation” to sensitize states to contrast the spread of misinformation (WHO, 2020). To date, one of the main problems consists in conspiracy news relating to alleged vaccine damage, which can seriously compromise the international strategy for the abatement of SARS-CoV-2 (Tollefson, 2021). Therefore, the demand for new effective and efficient infodemiological methods has never been as pressing as today. In this regard, scientists are increasingly adopting infoveillance tools to monitoring the infodemic on websites, social media, and newspapers (Zeraatkar and Ahmadi, 2020). Numerous research groups have exploited the state-of-art of machine learning to catalog and analyze the large flows of COVID-19-related information circulating on social networks, forums, and online platforms like Twitter, Reddit, Instagram, Facebook, and YouTube (Tsao et al., 2021). Among the most skillful approaches, Rustam et al. adopted a wide variety of supervised algorithms such as random forest (RF), XGBoost classifier, support vector classifier (SVC), extra trees classifier (ETC), decision tree (DT), and long-short term memory (LSTM) deep learning model to analyze COVID-19-related tweets sentiment (Rustam et al., 2021). Their results showed that: 1) Extra Trees Classifiers outperformed all other models by achieving a 0.93 accuracy score using the authors’ proposed concatenated features set; 2) the LSTM achieved low accuracy as compared to machine learning classifiers. Nonetheless, Jelodar et al. implemented a novel application for natural language process (NLP) based on an LSTM model for the same purpose on Reddit posts, obtaining convincing results (Jelodar et al., 2020). Mackey et al. also studied the dissemination of fake and dangerous information on Twitter and Instagram through NLP and deep learning (Mackey et al., 2020). Although this evidence seems contradictory, it merely shows the vast range of unexplored possibilities offered by machine learning for infodemiological aims and, at the same time, that the model accuracy depends strongly on the initial conditions. Ergo, Machine learning showed excellent effectiveness but it has limitations (Mohri et al., 2012). As highlighted by comparing scientific literature, its application needs ad-hoc interventions not always assimilable in a general methodology. Supervised algorithms require large training datasets to produce inferred functions for mapping new examples; such a procedure consumes time and resources, thus slowing down the infoveillance process consistently. On the contrary, unsupervized models learn from raw data without any prior knowledge; therefore, results might be less inaccurate and take more time if compared with supervised learning. Moreover, datasets always require appropriate processing before using them. In this regard, many authors have preferred to adopt more traditional methods, like multivariate regressions, cross-correlations, time-series analysis, and descriptive statistics (Tsao et al., 2021). The majority of these are now integrated into easy-to-use automatic kits available for Microsoft Excel software or similar (e.g., Real Statistics and Zaiontz, 2021; XLSTAT, 2021), which is a great advantage in terms of operational speed. However, when dealing with platforms such as Twitter, Reddit, Instagram, or Facebook, the collection and analysis of posts is still laborious: indeed, it requires the use of databases already extracted (which limits the power of investigation) or application programming interfaces (APIs) and all datasets must be suitably processed before use (Kim et al., 2020). Therefore, while all of the above methods are essential and powerful for historical data analysis, more immediate and rapid tools are equally necessary for quasi-real-time infoveillance. In particular, Google Trends—an open online infoveillance tool developed by Google™—has been widely used by the scientific community not only for monitoring disinformation but also for making rapid epidemiological predictions on the spread of infectious diseases (Mavragani and Ochoa, 2019). Google Trends quantifies the users’ web interest in a keyword (e.g., “football”) by returning a normalized value ranging from 0 to 100, called relative search volume, proportional to the ratio between the keyword-related queries and the total web queries. The user can also narrow the analysis to specific geographical areas (continents, states, regions, cities, etc.) in a fixed timelapse. In this regard, the quantitative analysis of relative search volumes of pre-selected queries was used for several purposes during COVID-19 pandemic: 1) predicting COVID-19 cases (Ahmad et al., 2020; Ayyoubzadeh et al., 2020; Jimenez et al., 2020; Mavragani and Gkillas, 2020; Sulyok et al., 2020; Venkatesh and Gandhi, 2020; Prasanth et al., 2021), 2) studying the web interest in COVID-19 (Effenberger et al., 2020; Hu et al., 2020; Rovetta and Castaldo, 2020; Springer et al., 2020), 3) studying the adoption of infodemic terms and related consequences (Cinelli et al., 2020; Cuan-Baltazar et al., 2020; Rovetta and Bhagavathula, 2020), 4) studying a full range of users’ psychological-emotional responses (Husnayain et al., 2020; Rovetta and Castaldo, 2020; Zattoni et al., 2020; Brodeur et al., 2021; Zitting et al., 2021), 5) studying the impact of mass media and governmental policies on users’ web searches (Rovetta and Bhagavathula, 2020; Sousa-Pinto et al., 2020; Huynh Dagher et al., 2021), 6) studying the economic-commercial impact (Brodeur et al., 2021; Sotis, 2021), 7) studying the spread of COVID-19 symptoms (Ahmad et al., 2020; Jimenez et al., 2020; Kluger and Scrivener, 2020; Walker et al., 2020), 8) studying other various web interests (Berger et al., 2021; Elsaie and Youssef, 2021). This type of research is mainly based on the search for statistical cross-correlations between users’ web searches related to specific topics, such as symptoms, drugs, therapies, vaccines, number of infected people, number of deaths, anxiety, fear, stress, etc., and the number of disease contagions and deaths officially registered after a certain timespan. However, not all that glitters is gold. First, many of these studies propose conflicting conclusions: specifically, some authors claim that the correlations between COVID-19 cases and web searches are generally spurious as mass media and government agencies’ announcements can influence them. Second, this paper shows that Google Trends has some limitations that are often overlooked and which risk heavily biasing and distorting correlation-based analytics. Furthermore, some anomalies in the calculus of relative search volumes (RSVs) could also alter any infodemiological analysis in an unpredictable way. Nonetheless, as shown above, a considerable portion of the academic world continues to rely on this tool to conduct its scientific investigations. This is probably due to the fact that Google Trends offers a simple and immediate way to obtain clean data (i.e., without complications related to privacy) on the vast majority of users’ web interests all over the world. This efficiency can be decisive in the epidemiological and infodemiological evaluation; indeed, although scientists and governments have launched mobile applications and websites with similar purposes (Kondylakis et al., 2020), the percentage of users involved is significantly lower than that of Google. Such services could also select a more targeted user, undermining the demand for randomness in the sample extraction (e.g., deniers and conspirators are automatically excluded from the dataset). Anyway, the combined use of both these methods could help us better understand their strengths and limitations and serve as a complete infoveillance approach. Therefore, the aim of this study is to delve into the aforementioned issues exploring their nature and searching for solutions to circumventing them, thus allowing the scientific community to continue using Google Trends through a more reliable approach.

## Methods

To assess the reliability of Google Trends (GT), relative search volumes (RSVs) of a specific query in a fixed period were downloaded on different days as to reveal any dependence on the date they were collected. According to Google, RSVs are calculated as follows: each data point is divided by the total searches of the geography and time range it represents to compare relative popularity. Otherwise, places with the most search volume would always be ranked highest. The resulting numbers are then scaled on a range of 0–100 based on a topic’s proportion to all searches on all topics (Google Support, 2021). In this context, “anomalies” were defined as those countries, regions, or cities whose RSVs appeared only on specific days.

### Data Collection

RSVs of the query *coronavirus + covid* were collected from two distinct periods: 1 February—4 December, 2020 (period 1), and 20 February—18 May, 2020 (period 2). As shown in previous studies, this query encompasses 80% of COVID-19-related web searches worldwide (Rovetta and Bhagavathula, 2020). For this reason, it has been considered well representative of the web interest in COVID-19. Furthermore, an independent verification confirmed these results, highlighting an increasing use of the keyword *covid* (Supplementary File S1). This final survey was carried out exploiting the rising queries and the associated queries directly provided by Google Trends. Period 1, corresponding to the Italian lockdown, was chosen for GT to provide daily RSVs, while period 2 was chosen for GT to provide weekly RSVs. The survey was carried out on Italian regions and cities, and worldwide countries and cities. All RSVs of periods 1 and 2 were collected daily for a minimum of 7 days and until any anomaly was highlighted; when no anomaly was identified within 15–20 days, the investigation was considered concluded. The data-collection period ranged from 8 to 25 December, 2020. The Google Trends category search-parameter was set to all categories. All details are shown in Table 1.

**TABLE 1:** Google Trends keywords summary.

Geographical region	Investigation period (2020)	Subregion	Collection period (2020)	Google trends URL^a^ ^,^ ^b^ ^,^ ^c^ ^,^ ^d^
Italy	February 1–December 4	Regions	December 8–26	URL Italy period 1
		Cities	December 14–26	
	February 20–May 18	Regions	December 8–26	URL Italy period 2
		Cities	December 14–26	
World	February 1–December 4	Regions	December 14–26	URL world period 1
		Cities	December 16–26	
	February 20–May 18	Regions	December 14–27	URL world period 2
		Cities	December 16–27	

a Google Trends 1, coronavirus + covid query in Italian regions and cities during period 1. URL: https://trends.google.it/trends/explore?date=2020-02-01%202020-12-04&geo=IT&q=coronavirus%20%2B%20covid.

b Google Trends 2, coronavirus + covid query in Italian regions and cities during period 2. URL:https://trends.google.it/trends/explore?date=2020-02-20%202020-05-18&geo=IT&q=coronavirus%20%2B%20covid.

c Google Trends 3, coronavirus + covid query in World countries and cities during period 1. URL: https://trends.google.it/trends/explore?date=2020-02-01%202020-12-04&q=coronavirus%20%2B%20covid.

d Google Trends 4, coronavirus + covid query in World countries and cities during period 2. URL: https://trends.google.it/trends/explore?date=2020-02-20%202020-05-18&q=coronavirus%20%2B%20covid.

Data on Italian COVID-19 cases was collected from the Italian Civil Protection Department official dashboard (ICPD, 2020). Data on international COVID-19 cases was collected from the World Health Organization official dashboard (WHO, 2020).

### Statistical Analysis

By calling i the country, region, or city under investigation and j the day its RSV was collected, a Gaussian distribution $X_{i}=X\left(\sigma_{i},{\bar{x}}_{i}\right),$ where $\sigma_{i}$ is the standard deviation (also called SD) and ${\bar{x}}_{i}$ is the mean value of $RSVs_{ij}$, was used to represent the trend of $x_{ij}=RSVs_{ij}$. To evaluate data normality, the Shapiro-Wilk test was used (Ghasemi and Zahediasl, 2012). The significance threshold was indicatively set at α=.05 (Amrhein et al., 2017). Data distributions that deviated greatly from α were marked with an asterisk (*). The impact of daily variations of $RSVs_{ij}$ in $X\left(\sigma_{i},{\bar{x}}_{i}\right)$ on Pearson (R) and Spearman (r) correlations with COVID-19 total cases was estimated; to do this, it was enough to compute the correlations on different days and calculate their percentage increases $\Delta =\left(u_{f}-u_{0}\right)/u_{0}\cdot 100$. For the adoption of these correlations, standard criteria were exploited (Mukaka, 2012). The Welch’s *t*-test $t=\left({\bar{x}}_{l}-{\bar{x}}_{m}\right)/\tilde{\sigma }$ (Kim, 2015) was performed in order to understand if the differences between the mean *RSVs* ${\bar{x}}_{l},\;{\bar{x}}_{m}$, extracted from the same geographical area and period but on different days, were significant. A difference between two RSVs was considered statistically significant when t>1.5. This test was considered appropriate since the mean values, together with their relative 95% confidence interval, well represented the samples (i.e. the arithmetic mean was sufficiently centered and the confidence interval comprised the clear majority of values). Furthermore, it does not require that the variances be similar. A dataset was deemed unreliable if the confident data exceeded 20% (confidence threshold) for at least one country, region, or city. When anomalies were identified in more than 20% of cases, no investigation on the distributions was conducted.

## Results

### Italian Regions’ Web Interest During Period 1 (1 February–4 December, 2020)

**FIGURE 1:**
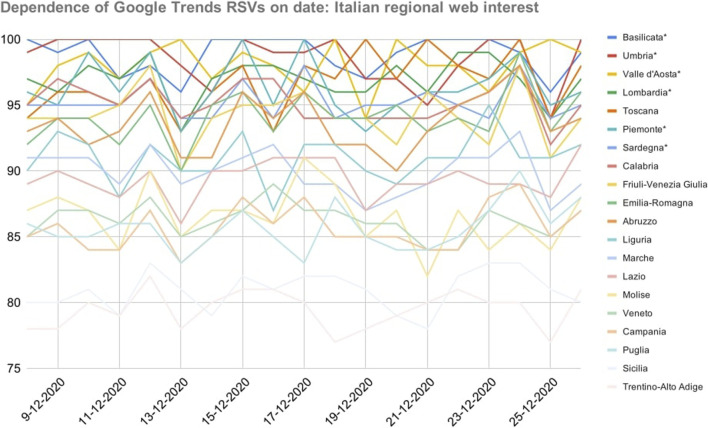
Dependence of Google Trends relative search volumes (RSVs) on collection date: Italian regions’ web interest in the query *coronavirus* + *covid* during period 1 (1 February–4 December, 2020). *X*-axis: dates on which the RSVs were collected. *Y*-axis: Google Trends RSV. * = Regions that showed a non-normal trend over time.

As shown in Figure 1, there have been strong relationships between RSVs and the dates they were collected: in fact, the regional ranking of web interest underwent several unpredictable variations even as regards the peak values *RSV* = 100.

The daily standard deviation of the sample ranged in the interval [6.6, 7.6], making all values in the central band mutually confident. Because of that, any correlations between RSVs and COVID-19 cases (or related statistics) could not be meaningful if merely based on a single-day dataset. Furthermore, even supposing no variance in daily samples, the correlation between the number of COVID-19 cases and RSVs went from r=−0.29 on December 8 to r=−0.36 on the following day (|Δ|=+24.1%). Considering the whole dataset, the same correlations ranged in the interval [−0.23, −0.42] (|Δ|=+82.6%). The mean value and standard error of the $X_{i}$ distributions were $\bar{x}=88.4$ and $\bar{SEM}\;=\;0.4$ respectively, with $SEM_{i}$ ranging in the interval [0.1, 0.7]. Therefore, the confidence threshold was exceeded (e.g., Abruzzo, 37%). However, no anomalies have been found.

### Italian Regions’ Web Interest During Period 2 (20 February–18 May, 2020)

**FIGURE 2:**
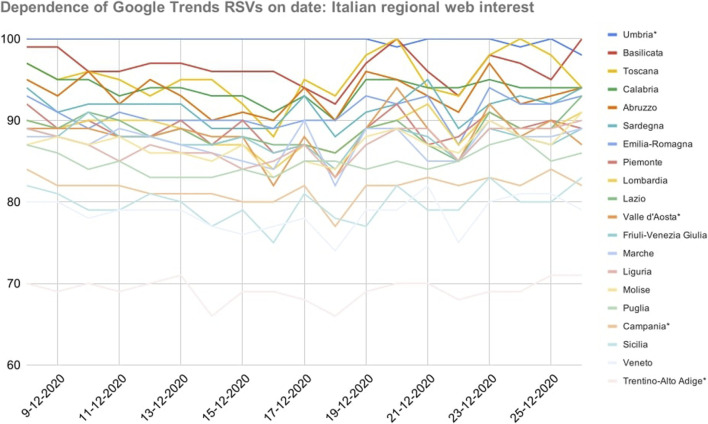
Dependence of Google Trends relative search volumes (RSVs) on collection date: Italian regions’ web interest in the query *coronavirus* + *covid* during period 2 (20 February–18 May, 2020). *X*-axis: dates on which the RSVs were collected. *Y*-axis: Google Trends RSV. * = Regions that showed a non-normal trend over time.

As shown in Figure 2 (next page), the variance of RSVs as a function of the day they were gathered was lower than that of the previous dataset $\left(\bar{x}=91.9,\;\;\bar{SEM}=0.4,\;\;{\bar{SEM}}_{i}\;\varepsilon \;\left[0.3,\;0.5\right]\right)$. This is probably due not only to the investigated period but also to the different sampling frequency. However, there was greater variability on RSV peaks and a larger number of non-normal trends.

The confidence threshold was exceeded (e.g., Abruzzo, 47%). Spearman and Pearson correlations between COVID-19 cases and daily RSVs ranged in the intervals [0.04, 0.29] (|Δ|=+625%) and [0.09, 0.26] (|Δ|=+189%) respectively. No anomalies have been found.

### Italian Cities’ Web Interest During Period 1 and Period 2

As shown in Tables 2, 3 (next two pages), significant anomalies occurred in 33.3% of Italian cities during period 1 and 45.8% during period 2. In particular, Perugia and Prato-absent respectively 7- and 10-times during period 1- recorded *RSVs* = 100 on 6 occasions. During period 2, Messina, Perugia, Pescara, Prato, and Salerno, recorded only 1 RSV out of 14 samples, while Parma recorded 2 RSVs. Therefore, any type of correlation or other statistical calculus, evaluation, or consideration on this dataset would be highly dependent on the day the data was gathered.

**TABLE 2:** Dependence of Google Trends relative search volumes (RSVs) on collection date: Italian cities’ web interest in the query *coronavirus* + *covid* during period 1 (1 February–4 December, 2020).

City	Weekly RSVs collected day by day from 14 to 26 December, 2020												
Bari	94	98	94	94	97	95	94	96	94	95	91	91	94
Bologna	94	92	96	94	96	95	95	96	95	94	90	91	94
Brescia	89	91	87	88	87	89	90	86	88	88	85	86	87
Cagliari	97	98	100	96	97	95	94	98	96	96	97	91	98
Catania	88	89	86	82	86	86	89	85	85	88	84	83	87
Firenze	96	100	98	100	100	97	100	99	97	98	100	95	97
Genova	88	92	89	91	89	91	89	92	90	90	89	86	87
Milano	89	94	90	90	91	91	91	91	86	93	90	88	90
Modena		95		92		95							94
Napoli	89	93	89	88	91	90	91	87	90	94	88	84	89
Padova	88	90	88	87	91	88	87	84	87	89	87	84	87
Palermo	79	84	79	80	81	80	78	78	80	78	79	79	81
Parma	87	88			89	87		86	85	86	87		
Perugia	100						98	100	100			100	100
Prato						100	100					95	
Reggio calabria								95		100	96		
Reggio emilia			88		90								
Roma	90	94	92	93	94	93	92	93	92	94	92	88	92
Salerno			87	86	87	84			85	88	85		85
Taranto						100							
Torino	92	92	95	91	96	88	96	90	87	97	88	88	87
Trieste	90	88	91	92	90	91	93	92	85	90	90	88	86
Venezia	82	85	83	81	80	83	81	82	79	84	80	79	80
Verona	83	85	86	81	86	86	86	84	82	84	82	79	82

**TABLE 3:** Dependence of Google Trends relative search volumes (RSVs) on collection date: Italian cities’ web interest in the query *coronavirus + covid* during period 2 (20 February–18 May, 2020).

City	Daily RSVs collected day by day from 14 to 26 December, 2020												
Bari	93	90	89	90	90	91	90	87	92	88	87	90	87
Bologna	96	95	96	95	95	96	95	92	95	92	92	98	95
Brescia	93	93	92	93	94	92	94	92	88	90	94	91	90
Cagliari	100	100	98		100	100	100	100	100	100	100	100	100
Catania	89	87	89	89	89	87	88	91	94	86	88	89	86
Firenze	93	93	95	96	95	96	96	97	97	94	93	96	93
Genova	88	87	86	88	89	89	89	86	89	83	85	89	86
Messina					77								
Milano	97	98	100	100	95	95	98	97	96	94	96	98	96
Modena					89			94	93	92	93		
Napoli	90	89	90	90	88	88	87	86	87	87	88	87	84
Padova	93	92	93		92	93	90	94	95	90	91	90	91
Palermo	78	77	79	77	77	79	81	79	79	75	78	78	74
Parma										83	86		
Perugia										97			
Pescara									95				
Prato												91	
Reggio emilia	87				86							84	
Roma	90	91	92	93	89	90	91	90	89	90	89	90	89
Salerno			86										
Torino	93	92	94	95	91	92	94	94	94	92	94	91	92
Trieste	90	92	90	91	86	89	91	89	89	89	86	89	89
Venezia	91	89	93	91	89	91	89	94	87	88	88	87	87
Verona				89		87	90		88		89		

### Global Web Interest During Period 1 (February–4 December, 2020)

Google Trends reported a maximum of 62 countries’ RSVs (Supplementary Table S1). Significant anomalies occurred in 6 cases (9.7%) and the peak RSV=100 was reached and maintained unchanged by Italy ($SD_{i}=0)$. In 64.5% of cases, data was not normally distributed. No nation exceeded the confidence threshold even if the dataset showed a high variability range if compared to that of Italy ($\bar{x}=43.0,\;\;\bar{SEM}=0.5,\;\;{\bar{SEM}}_{i}\;\epsilon \;\left[0,\;1.4\right]$). Spearman correlations with COVID-19 total cases ranged in the interval [0.04,0.11] (|Δ|=+175%); however, it must be pointed out that the value r=0.04 was an outlier (recorded on December 16, 2020) and a more representative interval is [0.10,0.11] (|Δ|=+10%).

### Global Web Interest During Period 2 (20 February–18 May, 2020)

Google Trends reported a maximum of 64 countries’ RSVs (Supplementary Table S2). Significant anomalies occurred in 7 cases (10.9%) and the peak RSV=100 was reached and maintained unchanged by Italy ($SD_{i}=0)$. In 56.2% of cases, data was not normally distributed. No nation exceeded the confidence threshold even if the dataset showed a high variability range if compared to that of Italy ($\bar{x}=44.5,\;\;\bar{SEM}=0.5,\;\;{\bar{SEM}}_{i}\;\epsilon \;\left[0,\;1.1\right]$). Spearman correlations with COVID-19 total cases ranged in the interval [0.04,0.11] (|Δ|=+175%); however, it must be pointed out that the value r=0.11 was an outlier (recorded on December 16, 2020) and a more representative interval is [0.04,0.06] (|Δ|=+50%).

### International Cities’ Web Interest During Period 1 and Period 2

As shown in Tables 4, 5 (next two pages), significant anomalies occurred in 30.4% of international cities during period 1 and 38.1% during period 2. In particular, Bogotà, Chicago, Dubai, Houston, Hyderabad, Los Angeles, Sao Paulo, Santiago of Chile were affected by anomalies during period 1 and period 2, which also included Milan (${\bar{RSV}}_{i}=100$) and Rome (RSV=100 on December 25, 2020). Therefore, any type of correlation or other statistical calculus, evaluation, or consideration on this dataset would be highly dependent on the day the data was gathered.

**TABLE 4:** Dependence of Google Trends relative search volumes (RSVs) on collection date: international cities’ web interest in the query *coronavirus + covid* during period 1 (1 February–4 December, 2020).

City	Daily RSVs collected day by day from 16 to 2**6** December, 2020										
Bangalore	60	63	63	59	63	60	62	63	61	62	61
Bogotá	48	49				49	50	49			
Chicago	62			63	62	62	62	63			64
Mexico city	50	49	50	49	51	51	49	49	51	49	50
Dubai										71	
Houston			52	54			51		53		
Hyderabad	43										
London	66	67	67	67	67	67	65	66	64	66	67
Los angeles	58	60	59	60	60	60	58	57		58	61
Madrid	80	82	82	85	81	84	80	78	80	80	84
Melbourne	87	88	86	88	84	87	85	83	85	85	88
Milan	97	100	98	97	100	100	94	100	100	97	100
Mumbai	73	74	70	71	69	72	72	71	72	71	72
New York	52	51	51	50	52	50	50	50	52	51	50
New Delhi	59	60	56	58	59	59	58	59	56	57	59
Paris	70	71	71	73	70	71	69	72	70	73	72
Rome	100	98	100	100	98	100	100	100	97	100	100
Sao paulo			32		33	33	34		34	33	
Santiago of Chile	43			44				44			44
Singapore	56	56	56	56	58	57	55	57	56	55	58
Sydney	61	60	61	60	59	60	60	58	60	61	61
Toronto	81	80	78	79	79	82	77	81	78	79	79

**TABLE 5:** Dependence of Google Trends relative search volumes (RSVs) on collection date: international cities’ web interest in the query *coronavirus* + *covid* during period 2 (20 February–18 May, 2020).

City	Daily RSVs collected day by day from 16 to 27 December, 2020											
Bangalore	67	67	65	67	65	66	66	66	70	68	65	65
Bogotá	50	52	49	51		50		51	52	53	50	48
Chicago	61	62	60			60		59	63	62		
Mexico city	46	46	45				48	46	47		46	
Houston	53	53	50		51	52						52
Hyderabad		49	48	49	48					50		
London	64	64	64	63	64	65	66	64	67	65	63	62
Los angeles	58	58	55	57	56	58	56	56	60	57	56	57
Madrid	83	85	85	83	82	85	86	84	84	86	83	87
Melbourne	60	61	60	58	58	60	59	58	62	64	58	60
Milan	100	100	100	100	100	100	100	100	100		100	100
Mumbai	78	77	75	76	76	76	76	78	78	80	77	77
New York	53	56	56	51	52	56	54	54	55	55	53	54
New Delhi	61	62	60	62	59	60	60	61	61	63	61	61
Paris	69	71	70	70	69	70	71	70	71	71	68	69
Rome	91	94	91		92		93	94	96	100	91	98
Sao paulo	34	34	32		33	35	35	32	36			33
Santiago of Chile	45		47		46		48				46	
Singapore	55	57	58	57	56	58	57	59	58	60	58	57
Sydney	55	56	56	56	54	55	55	56	57	59	55	57
Toronto	72	72	70	71	71	72	72	70	75	71	70	70

## Discussion

As far as the author knows, this is the first study to assess Google Trends reliability through an iterated queries analysis. In particular, this paper clearly demonstrates a strong dependence of Google Trends relative search volumes (RSVs) values on the date they are gathered. The dataset of Italian regions above all, although if not affected by anomalies, showed how the collection of the same queries’ RSVs (i.e. same category, area and period) on different days is able to substantially modify a statistical correlation between RSVs themself and an external quantity (in this case, the number of COVID-19 infections). Moreover, in all the other datasets, an even greater problem was highlighted such as the presence or absence of specific RSVs depending on the day the sample was gathered. This phenomenon has also affected cities that have reached peak values on several occasions, such as Milan and Rome in the global dataset and Perugia and Prato in the Italian dataset. Furthermore, the fact that Prato and Perugia have reached a peak of web interest in the Italian dataset but not in the international dataset shows how Google Trends RSV measurement includes only specific geographical areas according to the search item chosen by the user. Finally, RSVs of Italian regions and cities as well as RSVs of international cities showed such a daily variance that these areas were often statistically confident with each other, compromising any search for correlations or any other rank-based grouping. The most reliable dataset—i.e. a sample that showed an acceptable number of anomalies and whose data did not exceed the confidence threshold—was that of countries worldwide both during period 1 and period 2. However, even in this case there were outliers capable of destroying the correlation between RSVs and COVID-19 cases. The results of this research cast an aura of uncertainty in using Google Trends for making infodemiological or epidemiological evaluations. In all studies conducted so far, data was extracted only once as the authors could not expect the dependence of RSVs on the day of collection. Anomalies can disrupt the statistical significance of a correlation, as they can change the distributive nature of the sample by transforming it from Gaussian to non-Gaussian or vice versa. Thus, depending on the type of correlation, they can irrevocably compromise the use of *p*-values as graded measures of evidence against the null hypothesis. Moreover, since Pearson coefficient is sensitive to outliers, they can drastically affect its strength (Mukaka, 2012). Finally, pronounced changes in RSV can invalidate the reliability of a dataset. For example, no analysis on the geographical distribution of web interest in a chosen topic for a specific area can be carried out when RSVs varies significantly from day to day (although the investigated period is always the same). But these are not the only criticalities that this survey has pointed out: indeed, this evidence shows that any study performed through Google Trends is inherently not reproducible. At present, the actual algorithm by which Google Trends detects query data is unknown. This makes it difficult, if not impossible, to identify the causes of this phenomenon. Alongside the limitations highlighted in this work, Cervellin et al. pointed out that web queries can be influenced by main media, further reducing the credibility of this research tool (Cervellin et al., 2017). Nuti et al. have previously found that a large multitude of papers lack the information needed to make them fully reproducible (Nuti et al., 2014). Nevertheless, Google Trends has served and still serves as an excellent tool for infoveillance and infodemiology: in fact, even admitting that newspapers and newscasts can influence web queries, it provides a way to quantify the web interest in a specific topic more efficiently than any other methods historically used (e.g., population surveys) (Amber et al., 2016; Dreher et al., 2018; Mohamad and Kok, 2019; Havelka et al., 2020). Moreover, it can be used as a complement to a traditional analysis (Schootman et al., 2014). During the COVID-19 pandemic, it was widely used by the scientific community and continues to be. Therefore, infoveillance and infodemiology scholars must adopt a more robust criterion for collecting data from Google Trends. Specifically, a series of steps can minimize the likelihood of fatal misinterpretation: 1) the trend of the RSV of a query for pre-selected periods and geographic regions must be gathered and monitored daily to assess its stability, i.e. the absence of anomalies and dramatic changes in the RSV of geographic subunits (like cities, regions, or nations). 2) If the trend has been stable for at least 7 days, continue to download data until statistical incompatibility between the RSVs of the various subunits has been reached (e.g., Welch’s t-test > 1.5). If the dataset is not normally distributed, it is recommended to perform at least 30 extractions; otherwise, it is possible to adopt a non-parametric test. 3) Use the mean RSV values of each subunit as measures to represent the sample, also providing their 95% confidence interval (or variability range). 4) When searching for correlations between RSV and other quantities, calculate them for every daily dataset and provide each mean value together with its 95% confidence interval (or variability range). This technique refers to standard frequentist inference criteria, ergo it applies to any frequentistic dataset. The central limit theorem ensures that mean values and confidence intervals are valid statistical measures for making comparisons regardless of data distribution (Kwak and Kim, 2017). Regarding the influence of media or external sources on RSV, Sato et al. are developing an analytical approach to clean up data from these disturbances (Sato et al., 2021). This would consent to the adoption of Google Trends also in the epidemiological field. In conclusion, Google Trends represents a great source of information for the entire scientific community. Nonetheless, more details should be provided by Google on how RSVs are presented to users. To ensure full reliability of a Google Trends dataset, it is essential for future research that authors collect queries’ data for several consecutive days and work with their RSVs averages instead of daily RSVs, trying to minimize the standard errors until an established confidence threshold is respected. Anyway, since this analysis is limited to a single query in two fixed time frames, further research is needed to understand when and how the proposed method is sufficient to contain the oscillations of the RSV acceptably. In particular, it is necessary to establish the causal relationship between Google Trends datasets selection and the occurrence of anomalies and sudden changes in the RSV.

## Data Availability

The original contributions presented in the study are included in the article/Supplementary Material, further inquiries can be directed to the corresponding author.
