# Supplementary material for: Reliability of Google Trends: Analysis of the Limits and Potential of Web Infoveillance During COVID-19 Pandemic and for Future Research
Source: Front Res Metr Anal. 2021 May 25;6:670226. doi: 10.3389/frma.2021.670226 (PMC8186442; doi:10.3389/frma.2021.670226)
Supplement: Supplementary file 1 [file Table1.DOCX]

**Supplementary file 1**

| Category: all categories |  |  |  |  |  |
| --- | --- | --- | --- | --- | --- |
|  |  |  |  |  |  |
| Week | coronavirus: (Worldwide) | covid: (Worldwide) | sars-cov-2: (Worldwide) | cord-19: (Worldwide) | 2019-ncov: (Worldwide) |
| 2020-01-05 | < 1 | 0 | 0 | 0 | 0 |
| 2020-01-12 | < 1 | < 1 | 0 | 0 | < 1 |
| 2020-01-19 | 5 | 0 | 0 | 0 | < 1 |
| 2020-01-26 | 16 | < 1 | 0 | 0 | < 1 |
| 2020-02-02 | 9 | 0 | 0 | 0 | < 1 |
| 2020-02-09 | 7 | < 1 | < 1 | 0 | < 1 |
| 2020-02-16 | 6 | < 1 | < 1 | < 1 | < 1 |
| 2020-02-23 | 28 | 1 | < 1 | 0 | < 1 |
| 2020-03-01 | 35 | 2 | < 1 | < 1 | < 1 |
| 2020-03-08 | 75 | 8 | < 1 | < 1 | < 1 |
| 2020-03-15 | 100 | 19 | < 1 | < 1 | < 1 |
| 2020-03-22 | 83 | 24 | < 1 | < 1 | < 1 |
| 2020-03-29 | 66 | 22 | < 1 | < 1 | < 1 |
| 2020-04-05 | 68 | 20 | < 1 | < 1 | < 1 |
| 2020-04-12 | 58 | 19 | < 1 | < 1 | < 1 |
| 2020-04-19 | 43 | 17 | < 1 | < 1 | < 1 |
| 2020-04-26 | 31 | 16 | < 1 | < 1 | < 1 |
| 2020-05-03 | 28 | 16 | < 1 | < 1 | < 1 |
| 2020-05-10 | 24 | 16 | < 1 | 0 | < 1 |
| 2020-05-17 | 19 | 14 | < 1 | < 1 | < 1 |
| 2020-05-24 | 16 | 13 | < 1 | 0 | < 1 |
| 2020-05-31 | 14 | 12 | < 1 | < 1 | < 1 |
| 2020-06-07 | 13 | 12 | < 1 | 0 | < 1 |
| 2020-06-14 | 12 | 12 | < 1 | 0 | < 1 |
| 2020-06-21 | 13 | 14 | < 1 | 0 | < 1 |
| 2020-06-28 | 13 | 15 | < 1 | 0 | < 1 |
| 2020-07-05 | 13 | 15 | < 1 | < 1 | < 1 |
| 2020-07-12 | 12 | 16 | < 1 | < 1 | < 1 |
| 2020-07-19 | 11 | 15 | < 1 | 0 | < 1 |
| 2020-07-26 | 11 | 15 | < 1 | 0 | < 1 |
| 2020-08-02 | 9 | 14 | < 1 | < 1 | < 1 |
| 2020-08-09 | 8 | 14 | < 1 | 0 | < 1 |
| 2020-08-16 | 8 | 12 | < 1 | 0 | < 1 |
| 2020-08-23 | 7 | 12 | < 1 | < 1 | < 1 |
| 2020-08-30 | 7 | 12 | < 1 | 0 | < 1 |
| 2020-09-06 | 7 | 13 | < 1 | < 1 | < 1 |
| 2020-09-13 | 8 | 13 | < 1 | < 1 | < 1 |
| 2020-09-20 | 7 | 13 | < 1 | 0 | < 1 |
| 2020-09-27 | 7 | 13 | < 1 | 0 | < 1 |
| 2020-10-04 | 7 | 14 | < 1 | 0 | < 1 |
| 2020-10-11 | 7 | 14 | < 1 | < 1 | < 1 |
| 2020-10-18 | 7 | 15 | < 1 | 0 | < 1 |
| 2020-10-25 | 8 | 16 | < 1 | 0 | < 1 |
| 2020-11-01 | 6 | 15 | < 1 | < 1 | < 1 |
| 2020-11-08 | 7 | 17 | < 1 | 0 | < 1 |
| 2020-11-15 | 7 | 19 | < 1 | < 1 | < 1 |
| 2020-11-22 | 5 | 16 | < 1 | 0 | < 1 |
| 2020-11-29 | 5 | 15 | < 1 | 0 | < 1 |
| 2020-12-06 | 5 | 16 | < 1 | 0 | < 1 |
| 2020-12-13 | 5 | 17 | < 1 | 0 | < 1 |
| 2020-12-20 | 6 | 17 | < 1 | 0 | < 1 |
| 2020-12-27 | 6 | 18 | < 1 | 0 | < 1 |
| 2021-01-03 | 6 | 19 | < 1 | 0 | < 1 |
| 2021-01-10 | 5 | 20 | < 1 | < 1 | < 1 |
| 2021-01-17 | 5 | 18 | < 1 | < 1 | < 1 |
| 2021-01-24 | 5 | 17 | < 1 | 0 | < 1 |
| 2021-01-31 | 4 | 16 | < 1 | < 1 | < 1 |
| 2021-02-07 | 4 | 15 | < 1 | 0 | < 1 |
| 2021-02-14 | 4 | 14 | < 1 | 0 | < 1 |
| 2021-02-21 | 4 | 15 | < 1 | 0 | < 1 |
| 2021-02-28 | 4 | 16 | < 1 | 0 | < 1 |
| 2021-03-07 | 3 | 17 | < 1 | < 1 | < 1 |
| 2021-03-14 | 4 | 17 | < 1 | < 1 | < 1 |
| 2021-03-21 | 4 | 18 | < 1 | 0 | < 1 |
| 2021-03-28 | 4 | 19 | < 1 | 0 | < 1 |
| 2021-04-04 | 4 | 20 | < 1 | 0 | < 1 |
| 2021-04-11 | 4 | 19 | < 1 | < 1 | < 1 |
